# Supplementary material for: Thermooptical PDMS-Single-Layer Graphene Axicon-like Device for Tunable Submicron Long Focus Beams
Source: Micromachines (Basel). 2022 Nov 26;13(12):2083. doi: 10.3390/mi13122083 (PMC9788317; doi:10.3390/mi13122083)
Supplement: Supplementary file 1 [file micromachines-13-02083-s001.zip › micromachines-1995784-supplementary 2nd.pdf]

Supplementary Informations

1. Synthesis, transfer and characterization of graphene on glass

1.1 Synthesis of graphene by chemical vapor deposition (CVD)
Graphene samples were synthesized by low-pressure chemical vapor deposition (CVD) in a tubular quartz reactor. Cu foils (Alfa Aesar, order number 46986) were cut into ~1 cm^2 pieces, cleaned in a ultrasonic bath with acetone and isopropyl alcohol for 5 minutes, and dryed using N_2 gas to remove residual solvents. The Cu foils were inserted in the tube reactor under a constant flux of 50 standard cubic centimeters per minute (sccm) of H_2 gas. A temperature ramp of 30 °C min^{-1} from ambient temperature to 1050 °C was set using a tube furnace controller. The Cu was annealed for one hour at 1050 °C prior to the growth to reorient the Cu foils surface to (111) crystallographic direction [1]. After the annealing process, methane (CH_4) gas was introduced in the reactor at a flux of 5 sccm for twenty minutes with a total system pressure of 2.5 Torr. The reactor was cooled from 1050ºC to 650°C at a rate of 300 °C min^{-1} and then let to cool down to ambient temperature for the next hour under an H_2 flux of 50 sccm. After the growth, the Cu foils with graphene were stored in a vacuum chamber to avoid sample degradation.

1.2 Transfer of graphene to glass substrates
For the graphene transfer process, a polyurethane (PU) solution (1% molar concentration) was prepared with tetrahydrofuran (THF), and spin-coated on the Cu foils with graphene at 3000 rotations per minute (RPM) for 25 seconds. The foils were dissolved using an iron chloride solution, leaving the PU/graphene film floating on top of the solution. The floating PU/graphene films were transferred to a series of 3 baths of distilled water using 500 ml beakers. After cleaning the PU/graphene films with distilled water, the samples were transferred to the glass substrate and put on a hot plate for 10 minutes at 80 °C. Lastly, the Pu/graphene/glass samples were immersed in THF for 2 hours to remove the undesirable PU.

1.3 Raman characterization of single layer graphene samples on glass substrate
An NT-MDT (NTEGRA-SPECTRA) spectrometer equipped with an Andor CCD detector was used to acquire the spectra of the samples. A spectral resolution of 4 cm^{-1} is achieved with a 600 grooves/mm diffraction grating using a 473 nm solid-state laser as excitation light. In order to avoid sample damage and discrepancies in the spectra, several measurements were performed using a Mitutoyo M Plan Apo 100X objective lens with a numerical aperture of 0.7 and a neutral density filter array, yielding a laser spot of the order of 1 μm^2 with a incident power of less than 0.2 mW.

Figure S1 shows that Raman spectroscopy results are that of a typical single layer graphene transferred to borosilicate glass substrate. The I_{2D}/I_G ratio is higher than 2 [1, 2], and the I_D/I_G ratio shows that the defect density of the samples is low, indicating that the transferred samples did not sustain significant damages due to the growth protocol and transfer process [2]. Further, the Full width at half maximum (FWHM) of the 2D band at ~2700 cm^{-1} is ~25 cm^{-1}, also indicating a low concentration of point defects in the lattice, as well as that the line-shape of the peak is due to only one contribution, ruling out the existence of other scattering processes other than the two-phonon scattering with opposite momenta in the K point of the Brillouin zone [3,4].

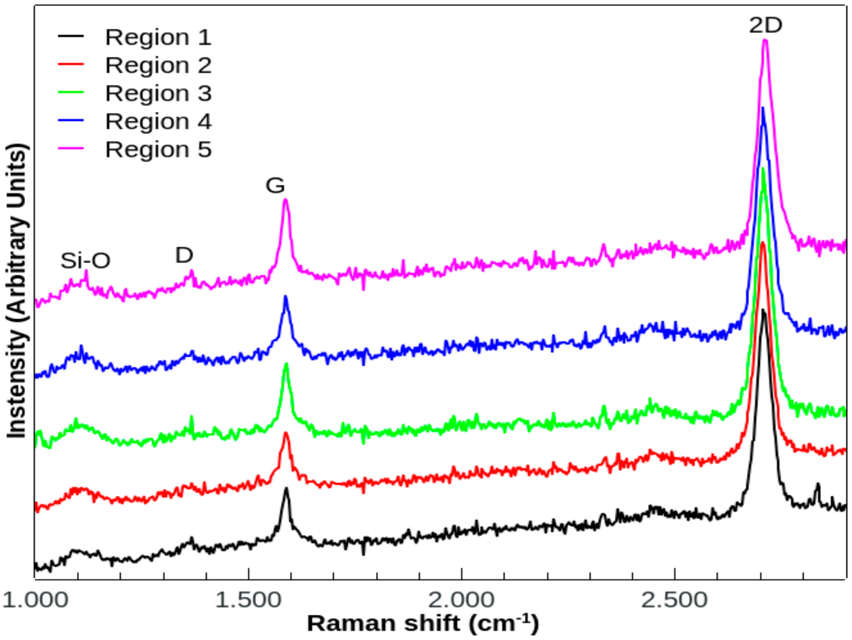

Figure S1: Raman spectra of transferred single layer graphene taken in different regions of the glass substrate. The mode at ~1100 cm^{-1} is due to the stretching of the Si-O bonds in the borosilicate glass. The D, G and 2D modes are typical of pristine single layer graphene positioned at ~1350 cm^{-1}, ~1580 cm^{-1}, and ~2700 cm^{-1} respectively.

2. Ultrafocusing with PDMS-SLG

The focus spot and focal line length are expected to reduce with the focal distance of Obj_1 . For the following evaluation we will consider the focal length of 0.9 mm of the commercial gradient index (GRIN) lens GT-LFRL-100-023-50-CC by Grintech. Considering the impinging Gaussian beam with a waist w_0=0.45 mm, at a wavelength of 405 nm, the theoretical focusing performances are resumed in the plots of Fig.S2 reported herebelow.

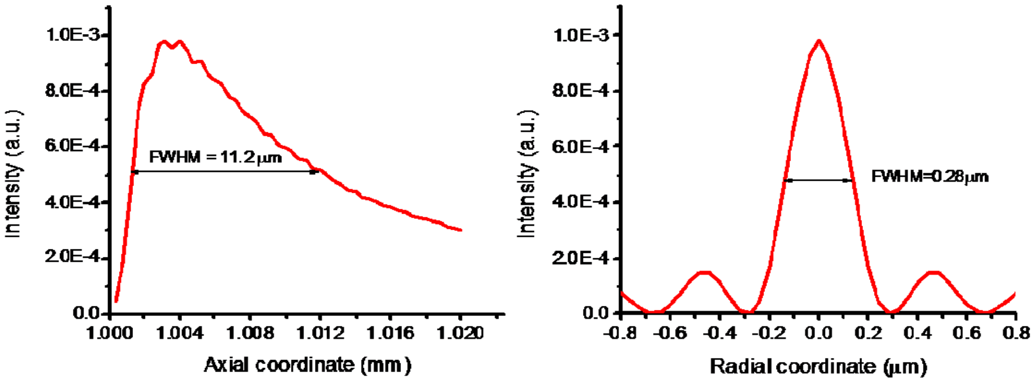

Figure S2 The focusing performances of a SLG-PDMS device coupled to a GRIN lens with o.9 mm focal length.

As shown, the FWHM of the spot focus focus is 0.26 μm, that increases to 0.315 μm at 532 nm wavelength, 80 nm higher (+31%) than that one, 260 nm, demonstrated in (aaa) at this wavelength. However, as described in the main text, in some applications the depth of focus is more significant than the spot diameter, and in our case it is 11.2 μm, of the same order of that one reported in the same reference. It is worth to notice that the use of micro lenses to focus the probe beam would greatly help to design a very compact and dimensions reduced long focus forming device.

References

1. Do Nascimento Barbosa A.; Figueroa J.S.; Mendoza C.D.; Pinto A.L.; Freire Jr. J.L. Characterization of graphene synthesized by low-pressure chemical vapordeposition using N-Octane as precursor. *Materials Chemistry and Physics*, **2018,** *219*,189-195.

2. Mastrapa Capote Gil; Freire F.L., Plasma-treated CVD graphene gas sensor performance in environmental condition: the role of defects on sensitivity. *Journal of Sensors*, **2019**, 2019.

3. Barbosa A. N. et al. Ptak F.; Mendoza C.D.; Maia da Costa M.E.H.; Freire F.L. Jr. Direct synthesis of bilayer graphene on silicon dioxide substrates. *Diamond and Related Materials*, **2019**, 95, 71-76.

4. Ferrari A.C.; Meyer J.C.; Scardaci V.; Casiraghi C.; Lazzeri M.; Mauri F.; Piscanec S.; Jiang D.; Novoselov K.S.; Roth S. Raman spectrum of graphene and graphene layers. *Physical review letters*, **97**, 18, 187401.
